# Theoretical Basis for Refractive Index Changes Resulting from Solution Phase Molecular Interaction

**DOI:** 10.1021/acs.jpcb.4c07563

**Published:** 2025-03-25

**Authors:** Michael N. Kammer, Amanda K. Kussrow, Darryl J. Bornhop

**Affiliations:** Department of Chemistry and The Vanderbilt Institute of Chemical Biology, Vanderbilt University, Nashville, Tennessee 37240, United States

## Abstract

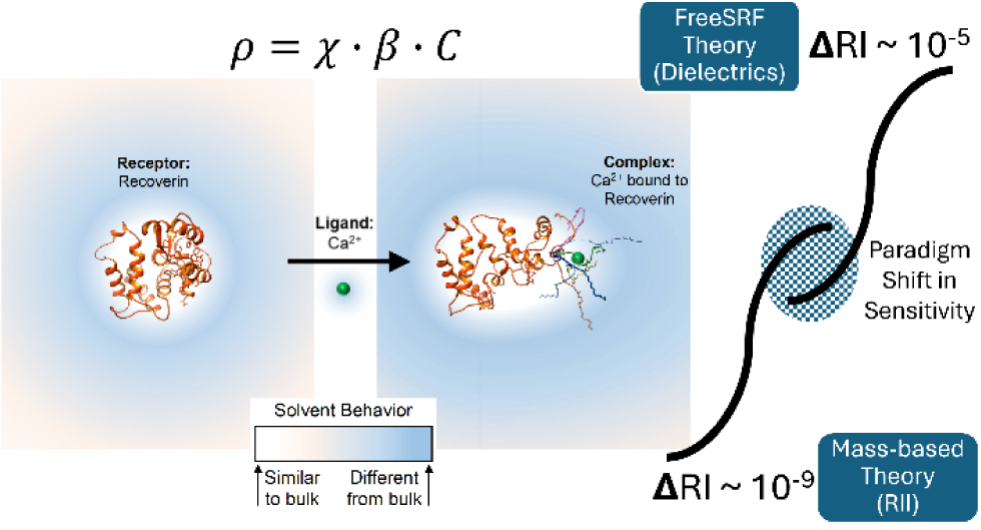

Refractive index (RI) is a fundamental optical property widely used to investigate the physical and chemical characteristics of materials. Here, we build on our previous work to refine the framework for RI sensing in solution-phase chemical and biochemical interactions. Starting from the Clausius–Mossotti relation, we present a first-principles derivation of a relationship for the RI signal resulting from chemical binding. We then demonstrate how the binding-induced conformational and hydration changes of interacting species relate to their estimated change in dielectric and thus the solution-phase change in refractive index (ΔRI). By varying the model parameters, such as solvation shell size and polarizability, we investigate the RI changes for two interactions: Ca^2+^ with the protein Recoverin and benzenesulfonamide with carbonic anhydrase 2 (CAII). These examples show that our theory predicts that even for small changes in binding-induced *polarizability* (relative to previous literature values), a quantifiable RI change is produced within the detectable range of RI detectors operating at ca. 10^–6^ RIU. Empirical observations confirm our theoretical predictions. Surprisingly, theory and experiment yield a *decrease* in ΔRI for the benzenesulfonamide-CAII interaction. We attribute this observation to shielding of charged residues and water molecule displacement during the binding event. Our approach is generalized, enabling it to be extended to other binding systems, as well as those undergoing nonbinding conformational changes, and facilitates the exploration of diverse biological and chemical processes by solution-phase RI sensing.

## Introduction

The refractive index (RI) is a fundamental optical property of materials.^1^ Therefore, understanding how light propagates through a material by measuring the RI can provide insights into the physical and chemical properties of the sample. The first laboratory instrument to accurately measure the refractive index of liquids was developed by Ernst Abbe in 1874^2^ and in the years since, the RI measurement has been a staple for determining the bulk properties of a sample. RI detectors take on various forms, ranging from simple deflection techniques to the ultrasensitive approaches represented by interferometry. RI measurements are considered “universal” providing qualitative and/or quantitative information about a sample and having been widely used in separation science and food processing.^3−6^ When properly implemented, the readout produced by an RI measurement can provide critical chemical and biochemical information such as solute/analyte concentration,^7−9^ reaction progression,^10^ molecular structure,^11,12^ and a measure of equilibrium binding or interaction affinity.^13−16^ Recently the free-solution method has been used to study allosteric modulators of lysophosphatidic acid 1 (LPA1),^17^ electrostatic interactions between Rhodopsin Kinase GRK1 and recoverin,^18^ and heparin binding to an engineered virus-like nanoparticle antagonist.^19^

The focus of this study is to further demonstrate that interferometric-based RI sensing can be used to interrogate interactions of chemical/biochemical species (proteins, small molecules, ions, etc.) *in solution*. We address the emerging need to understand solution-phase protein interactions, an area of research with wide-ranging implications, from drug development to further defining the processes of life. Herein we present a first-principles-based derivation of a relationship for the change in refractive index signal due to solution-phase intermolecular interactions based on the Clausius-Mossotti relation. Results from two experimental systems provide validation of our theory, demonstrating the magnitude of reaction induced signal as ΔRI is considerably larger than previously thought possible (ca. 10^–4^–10^–6^ vs 10^–8^–10^–10^ RIU).^20,21^ As a result, limits of detection for quantitative assays are typically picomolar and can reach femtomolar, without a relative mass dependence for interacting species. Finally, we discuss how our model and interferometric RI measurements can inform about physical and chemical behavior, such as the extent of binding-induced conformation and hydration changes in the participating species. Such observations are important toward defining the mechanism of action, particularly relevant for drug development. These observations can also be used to expand our fundamental understanding of solution-phase chemistry with respect to hydration shell size and the electrostatic properties that impact molecules (particularly proteins) in solution. As discussed here, hydration shell properties are important toward predicting binding-induced solution polarizability changes (RI). As many have suggested water plays a key role in complex stability and defining the propensity for species to bind (interact).

Recent work in our lab and others shows that performing RI measurements using a volume-constrained compensated interferometer allows for the transduction of molecular binding into assays that elucidate mechanisms of action,^13,22,23^ development of quantitative assays^8,9^ and validation of biomarkers of disease.^24,25^ These Free Solution Assays (FSAs) can be performed without the requirement to modify the constituent species through the addition of labels (radioligands or fluorophores) or through immobilization to a surface or bead. We have also demonstrated the magnitude and direction of the assay RI response is driven by conformational/hydration changes upon binding, allowing informed probe design aimed at S/N optimization.^26,27^

RI is a macroscopic property that emerges from the dielectric constant (k) of a solution. The dielectric constant is a bulk property that reflects the sum of the dielectric distribution across the path-length of electromagnetic radiation through a medium. Since k describes the permittivity of electromagnetism at any point in space, it depends on the polarizability of all the atoms within the medium and is related to the ability of an electromagnetic wave to deform the electron cloud around these atoms. Furthermore, the level of electron cloud deformation is a result of the electronic structure and electromagnetic forces, such as covalent bonds, hydrogen bonds, and van der Waals forces, in addition to kinetic forces arising from temperature and pressure. Therefore, it is our supposition that any theory of solution phase ΔRI measurement must account for changes in the polarizability, and therefore the dielectric, of the participating species (molecules) present in the solution.

Our previous efforts to understand the physical basis of FSA signal transduction by an interferometer (RI detector) resulted in relationship analogous to Beers Law termed the Free-solution Response Function (FreeSRF).^12^ FreeSRF is an experimentally derived relationship that defines the interferometric signal for a label-free, free-solution binding-induced change in solution-phase RI. FreeSRF_EXP_ for a binding pair is expressed in terms of the RI sensor sensitivity, the magnitude of conformation and solvation change during binding, the change in RI, and the concentration of the *product* of the interacting species. FreeSRF_EXP_ is defined as
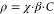
1where ρ is the signal magnitude measured in radians (e.g., refractive index units, RIU), χ is the Molar Refractometry in RIU/mol/L, which represents the relationship between ΔRI and the change in conformation and solvent accessible surface area, β is the instrument response function in radians/RIU, and C is the concentration of the reaction *product* in mol/L. We posit that ρ, originally derived in units of radians, could take the form of any sensible physical output of a refractive index detector. For example, while the interferometric signal is measured in radian shift, the output signal on a matched-bicell-based refractometer would be in voltage across the bicells. Thus, ρ would have units of volts, while the instrument response function β would have units of Volts/RIU.

In the simplest terms, FreeSRF_EXP_ is a heuristic approach that relates the change in RIU to changes in the molecular conformation and solvation upon solution-phase binding. The model has enabled a description of FSA that allows the accurate prediction of the magnitude and direction of binding induced RI changes based on structure and hydration changes.^12^ The approach has been validated on numerous analyte pairs with wide ranging relative masses, including ion-protein, protein–protein, protein-small molecule, protein complex-protein or small molecule, DNA hybridization, RNA-DNA and DNA aptamers with small molecules or proteins. While powerful, FreeSRF_EXP_ is not without limitations: as a heuristic model, there was no direct connection to solution-phase electromagnetic permittivity. Here we provide a first principals derivation of FreeSRF and then demonstrate how an enhanced consideration of the importance of solvation can further strengthen the model collectively enabling a deeper understanding of fluid-phase properties.

## Results and Discussion

### First Principals Derivation of FreeSRF

To allow for revision and further validation of FreeSRF, we start our derivation with the Clausius-Mossotti (CM) relationship, eq 2, (a consequence of Maxwell’s Equations)^28,29^ which relates the RI (*n*) of a dense medium to the sum of its molecular polarizabilities:
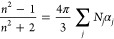
2where *n* is the refractive index of the medium, *N* is the number density (concentration) of interactants and α is the polarizability of all participating molecules in cubic angstroms (Å^3^).

We define the two unbound species that interact as the ligand and receptor, and the result of the interaction as the *product.* Considering α, *N*, and n before and after binding-induced changes, we can write two discrete CM equations to describe the RI for the solution. One equation describing the RI of the solution consisting of unbound ligand and receptor and the other for the RI of the solution containing the product.
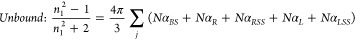
3
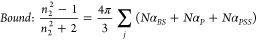
4

Where α_BS_ is the polarizability of bulk solvent, α_R_ the polarizability of the receptor, α_RSS_ the polarizability of the receptor’s solvation shell, α_L_ the polarizability of the ligand, α_LSS_ the polarizability of the ligand’s solvation shell, α_P_ the polarizability of the product, and α_PSS_ the polarizability of the product’s solvation shell. Subtracting eq 3 from eq 4 gives a relation that represents the change in RI (*n*_2_*– n*_1_) resulting from a chemical/molecular interaction:
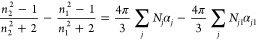
5

Bulk solvent is defined as solvent sufficiently removed from the surface of the *solute* to not be impacted by the solute. By definition, it remains unchanged by the interaction and therefore is subtracted from the expression (i.e., *N*_*b*1_*α*_*b*1_ = *N*_*b*2_*α*_*b*2_, where *N*_*b*_ is the number of bulk waters and *α*_*b*_ is the polarizability of a bulk water molecule). As a point of reference, it has been demonstrated that solvent molecules can be affected by the presence of solute out to ∼50 nM from the surface of the molecule.^30−32^ For the sake of this derivation, the distance of nonparticipating solvent is considered arbitrary, with several cases being considered below.

Treating the solvation shell as a single molecular entity, for 1:1 binding, for every 1 receptor (*N*_R_) there will be 1 ligand, receptor, product, and each of their solvation shells (*N*_R_ = *N*_*RSS*_ = *N*_L_ = *N*_LSS_ = *N*_*P*_ = *N*_PSS_, where *N*_*P*_ is the number of complexes and *N*_*PSS*_ is the number of the complex solvation shells). Functionally, this would mean that for a given molecular species, all waters in the solvation shell are considered a single entity, whether this includes tens of water molecules (as in the case of an ion) or many thousands of water molecules (as in the case of a large molecule). This entity will have a polarizability value of α′, where *α*_*SS*_ = *N*_*water*_ × α_water_, where *α*_*water*_ is the polarizability of a single water molecule within the solvation shell and *N*_*water*_ is the number of waters in the solvation shell. Solvation shell size and/or density changes or binding induced polarizability of water molecules within the solvation shell can be reflected as *α*_*RSS*_ + *α*_*LSS*_ ≠ *α*_*PSS*_ for those water molecules. Any receptor or ligand that remains unbound following a reaction will have the same polarizability before and after the interaction, so their contribution can be subtracted out. Therefore, the right-hand side of eq 5 simplifies to *N* · Δα, where N is the concentration of the product and the change in polarizability is Δα = (α_*P*_ + *α*_*PSS*_) – (α_R_ + α_*L*_ + *α*_*LSS*_). Simplifying eq 5 (as detailed in the supplemental info) provides an expression for ΔRI (Δn):
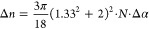
6

Collecting the constants into a single term *k*, we obtain
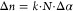
7

Equation 7 relates the solution phase change in RI, *Δn*, to the number density of conformational change events (*N*) (concentration of molecules that undergo a conformational rearrangement due to binding), the change in molecular polarizabilities (Δα) due to the conformational/hydration change, and a collection of constants (*k*, which is approximately 7.469). Equation 1 (FreeSRF_EXP_) and eq 7 (FreeSRF_CM_) are equivalent in that they both relate the change in measured binding signal or ΔRI, (ρ and Δ*n*), to a change in conformation and hydration (χ and Δα), the concentration (C and N) of the product, and a collection of constants (β and *k*). Thus, we have shown that a form of FreeSRF can be derived from first principles.

### Evaluation of the FreeSRF_CM_ Expression and the Importance of Solvation

Past attempts to reconcile free-solution Δ*n* signals with theory have focused primarily on the interacting species. However, examination of relevant literature clearly demonstrates that the volume of solvent affected by the solute is significantly larger than the solute itself.^33^ For example, a spherical 50 kDa protein will have a diameter of ∼2.4 nm, resulting in a volume of ca. 58 nm^3^.^34^ However, if the solvation shell extends just 4 nm,^32^ the volume of water within this solvation shell will be approximately 1100 nm^3^, nearly 20-fold larger than the volume of the protein alone. Additionally, water is highly polarizable, with an average polarizability of a water molecule within the solvation shell of a biological molecule of 1.5 Å^3^ per water molecule, which has a volume of 25 Å^3^.^35,36^ In other words, water has a polarizability of 1.5/25, or a polarizability ratio of 0.06. Comparatively, the polarizability of protein is approximately 0.0084, meaning that water is nearly 10-fold more polarizable than protein, on average. *Taken together, these observations demonstrate the important role of the solvation shell in the properties of the solution dielectric.*

“Solvation shell” waters have notably different densities and dielectrics than waters within the bulk solvent, thus they behave much differently in response to electromagnetic radiation than do bulk waters. The role of hydration layer size and extent of physical perturbation on solution dielectric is of great importance and has received heightened interest.^30,31,37−39^ Simply put, understanding solvation is key to better interpreting solution-phase interactions and in recognizing why the solution phase assay (FSA) measurement can transduce a wide array of interactions with high sensitivity.^12^ In the broadest sense, solution phase interactions are ubiquitous, driving essentially all chemical and biochemical processes.^40^ They are also key to many sensor technologies, such as isothermal titration calorimetry and fluorescence resonance energy transfer. For our purposes, accurately defining the magnitude of the solvation shell and its contribution to binding induced RI changes provides insight into the FSA mechanism and should enable FreeSRF to be predictive. Here we will consider three factors that likely impact Δ*n* signal magnitude in FSA: 1) the volume of the solvation shell, 2) the dielectric distribution within the solvation shell and 3) how the solvation shell differs from “bulk” water with respect to the dielectric/polarizability.

With eq 7, we can evaluate the relationship between the volume of the solvation shell, the magnitude of RI change for each interacting pair of molecules and determine if the solution-phase RI signal would be measurable. For clarity, we delineate 3 terms: 1) the Free-solution Response Function (FreeSRF) refers to the general theory that states binding-induced changes in conformation and hydration in free-solution produce a change in the solution’s RI. 2) The experimentally derived Free-solution Response Function (FreeSRF_EXP_) refers to the heuristic model for predicting the magnitude of Δ*n* from the radius of gyration and solvent-accessible surface area (for a known structural change), obtainable from quality X-ray crystallography or NMR structures. 3) The Clausius–Mossotti Free-solution Response Function (FreeSRF_CM_), is the mathematical expression derived from first-principles and relates change in solution-phase polarizability (and refractive index) to molecular rearrangement and changes in solvation.

Calculating the exact solution-phase molecular polarizability for biological macromolecules is a challenging and computationally intensive problem.^41^ However, the polarizability of the receptor and ligand can be estimated from structural information, and then the range of Δα produced by a binding event can be estimated reasonably by using data presented in the literature. With these parameters in hand, it is straightforward to estimate the ΔRI of a binding event and is done by estimating Δ*n* produced over a defined range of Δα. Obtaining the predicted Δ*n* in this manner allows for the comparison of the FreeSRF_CM_ model with experimentally measured values of Δ*n* and represents a major step in theory validation.

Due to computational complexity we cannot yet predict the polarizability of the product (*α*_*P*_) *de novo* from the polarizability of the receptor and ligand (*α*_*R*_*+ α*_*L*_), yet we can simplify the analysis by considering the ratio of the polarizability of the product to the sum of polarizability for the receptor and ligand. This ratio is denoted by the parameter Γ.
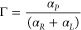
8

Per eq 8 Γ represents the ratio of the product’s *polarizability* (*α*_*P*_) to the sum of the *polarizability* of the unbound receptor (*α*_*R*_) and ligand (*α*_*L*_). When Γ = 1, there is *no change* in *polarizability* upon binding. In fact, setting Γ = 1 makes *no* physical sense for any molecular interaction, because one or both constituents will have its solvation shell altered due to the binding interaction. The more relevant cases are a) for Γ > 1, or a net increase in *polarizability* which leads to a positive RI change, or b) for Γ < 1, or an overall decrease in polarizability, resulting in a negative change in solution RI. Both scenarios have been experimentally observed and described.^42−44^

Rearranging eq 8 gives the expression for the change in polarizability of the product, Δα_p_ = (α_R_ + α_L_) ·(Γ – 1). Substituting this expression into eq 7, incorporating Avogadro’s number (N_A_) to convert concentration into number density and applying a volume conversion of 10^–27^ L/Å^3^, we obtain eq 9.
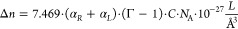
9

This expression lets us calculate the change in RI (Δn) for an interaction, as a function of the product molar concentration (*C*) and the change in polarizability of the participants (receptor and ligand). To obtain the value for α_R_, the polarizability of the receptor and its solvation shell are calculated and summed (detailed in the methods section). In short, protein polarizability is calculated by summing the contribution to polarizability of each amino acid,^45^ based on values for amino acids reported by Voges and Karshikoff.^46^ Solvation shell polarizability was obtained by calculating the volume of the solvation shell from the radius, varied over a range as defined below and based on literature values of solvation shell extent (as described in the Supplemental Methods). We then divide the solvation shell volume by the volume of a single water molecule to obtain the approximate number of waters within the solvation shell. Then multiplication by 1.5 Å^3^ per molecule, the *average* polarizability of a water molecule within a protein solvation shell^35,36^ we arrive at an estimate of α_R_.

For small molecule ligands, or any moiety not comprised of amino acids, the polarizability is calculated by using an atom-based composite model, where the polarizability of each atom within the molecule is added together.^47^ A detailed stepwise description of this calculation is presented in Figure S1.

Based on work performed by Ball and Ramsden,^48^ Γ for a solvation effect can range from 0.56 (the polarizability of the final conformation is only 56% of the polarizability of the initial conformation) to 1.77 (the polarizability of the final conformation is 77% larger than the polarizability of the initial conformation). While these values are derived from a single protein system with only a handful of results, it does allow reasonable bounds for Γ to be estimated. Similar ranges for protein refractive index depending upon solvation and conformation have been reported by others. As early as 1935, Timasheff showed that the measured d*n*/d*c* could vary based upon solvation effects: serum globulin’s dn/dC can range from 0.00146 to 0.00230 based upon solvent, suggesting a Γ from 0.63 from smallest to largest, or 1.57 from largest to smallest. Timasheff reported similar ranges for several proteins, including Gliadin, Casein, and serum albumin. Khago et al. described how measured dn/dC for various proteins was between 10 and 20% higher than would be calculated based on amino acid contribution alone. These observations are consistent with a Γ of 1.1 – 1.2 based simply upon solvation effects.^48−51^

We now explore the prediction of Δ*n* for two test systems using FreeSRF_CM_ for a range of Γ. The systems are Calcium ion binding Recoverin and Benzenesulfonamide binding Carbonic Anhydrase II. First, we explore the Δ*n* for a generalized scenario, one where there is *little information* available for the solvation shells of the participating species. Next, we show how information about the binding interaction, such as knowledge of structural rearrangement upon binding, can better inform the range of predicted Δ*n* estimated by FreeSRF_CM_.

**Figure 1:**
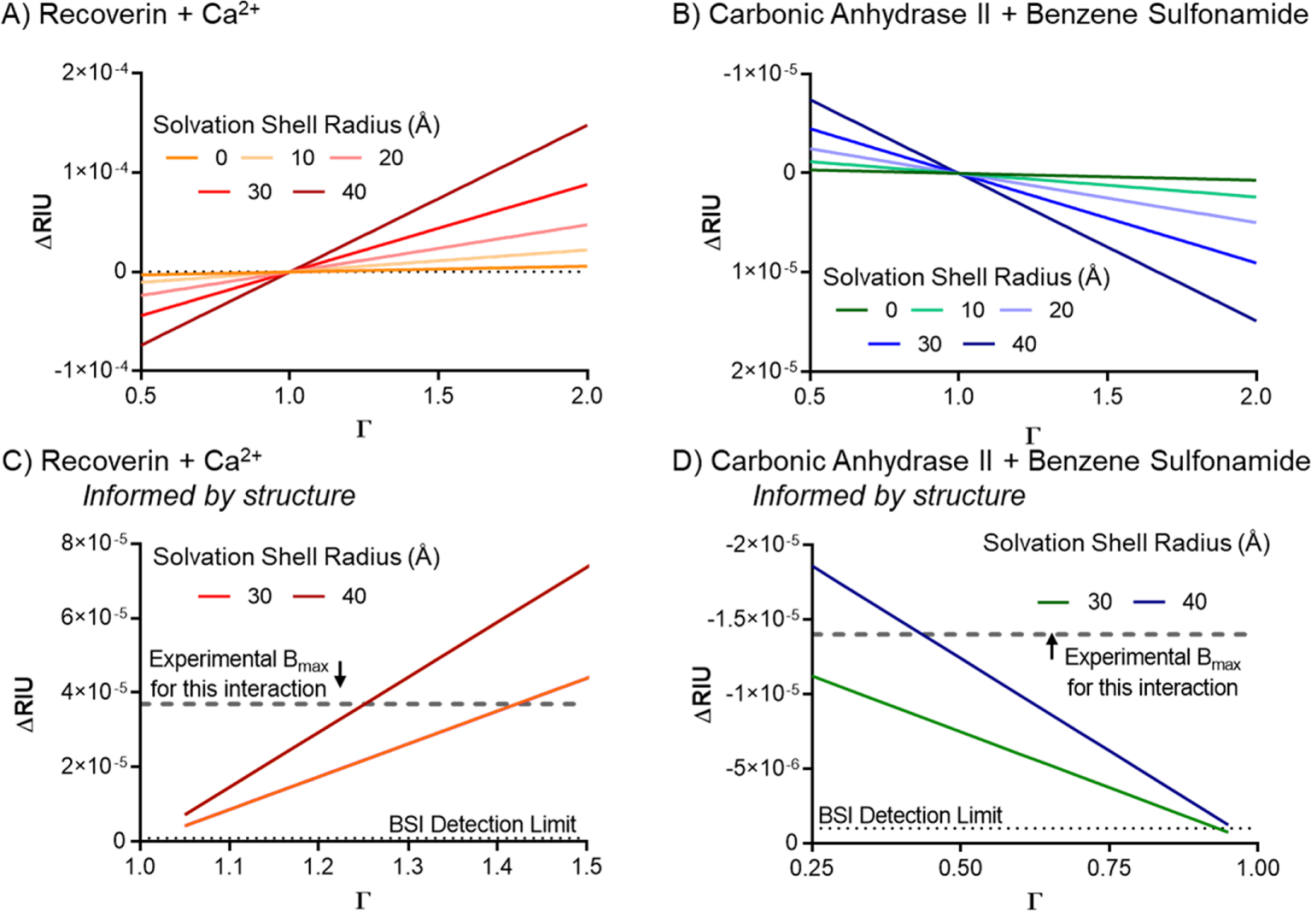
Predicted free-solution binding induced RI change for a range of Γ values and solvation shell sizes. Calculated from eq 8, derived from the Clausius-Mossotti equation. (A) Recovering binding calcium ion. (B) Carbonis Andydrase II binding benzenesulfonamide. The range of Γ can be informed by structural information on the binding system for a more defined calculation (C and D), shown here using a 30Å (light orange in C, green in D) and 40Å (dark red in C, dark blue in D) solvation shell, overlaid with the experimental Bmax for each binding event and the BSI detection limit. Note, the Y axis for B and D is reversed due to the decrease in polarizability.

Using the example where the solvation shell is not well-defined, we estimate Δ*n* over a significant range of Γ for a range of solvation shell radii with values from 0 to 40 Å. Radii were chosen based upon literature values for the extent of the solvation shell.^30,31,37−39^Figure 1A,B shows the results of these predictions for the two binding pairs evaluated: Recoverin binding Ca^2+^ (**1A**) and Carbonic Anhydrase II binding Benzenesulfonamide (**1B**). These plots illustrate that for essentially any sensible solvation shell size, e.g., from 5 to 40 Å, Γ needs to be only *slightly different from 1* to produce a Δ*n* large enough to be detectable with a refractive index detector exhibiting a sensitivity of ∼10^–6^ RIU. Even for the most conservative estimates of solvation shell size (∼10 Å, much lower than the solvation shell sizes typically reported for proteins,^31,37,38^ and small change in Γ of just 5%, results in a FreeSRF_CM_ RI signal of 1.2 × 10^–6^ RIU predicted for the Recoverin + Ca^2+^ interaction. This RI signal is detectable by both interferometric methods and many standard benchtop refractometers.

**Figure 2:**
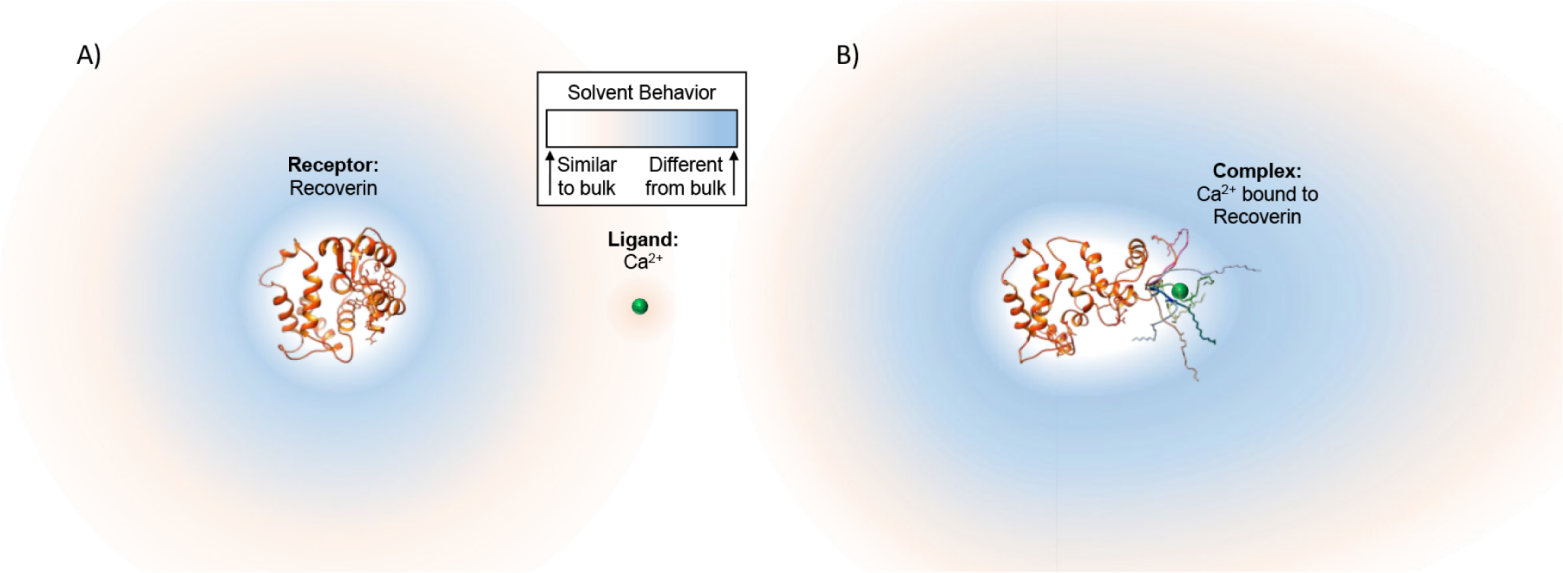
When recoverin binds to Ca^2+^, a buried myristoyl chain is exposed and leads to a more disordered protein (see structures in A and B). (A). The unbound receptor perturbs the solvent around it (the solvation shell). This perturbation is more pronounced near the protein and diminishes with the distance from the protein (blue and orange shaded areas). The unbound ligand perturbs the solvent in a similar way. (B). Since the myristoyl chain is disordered, the distribution of polarizability in the solvation shell is changed.

Importantly, information on binding-induced conformational rearrangement can be used to refine the range of Γ, and thus improve the estimation of Δ*n*. For example, Figure 2 shows that when a Ca^2+^ ion binds the neuronal protein recoverin, a buried myristoyl chain is exposed. This chain has considerable flexibility and can undergo significant conformational rearrangement. Molecular dynamics simulations and tetrahertz spectroscopy experiments confirm that disordered protein structures exhibit solvation shells with increased polarizability relative to their ordered form.^33,37,52,53^ Therefore, it stands to reason that a conformation change should result in a large change in the distribution of polarizability of water within the solvation shell (blue halo in Figure 2A,B). For recoverin interacting with Ca^2+^, which represents an extremely small relative mass change, the overall result of this structural change and water redistribution is a relatively large, positive, binding-induced change in solution-phase *polarizability*.

Figure 1C (**dark red line**) shows the results of taking a conservative approach of varying Γ from 1.05 (small, 5% polarizability increase) to 1.5 (a 50% polarizability increase) for a solvation shell of 40 Å for the Recoverin-Ca^2+^ interaction. This change in polarizability is less than the largest change seen in Ball and Ramsden’s experiment which explores the buffer dependence of RII of protein solutions,^48^ those reported by Hand’s work on evaluating protein solutions in various solvents,^49^ and similar in order of magnitude as the work reported by Khago et al. for the difference between protein composition and conformation.^51^ In this example, for a 540 nM Recoverin/Ca^2+^ product concentration, the predicted Δ*n* ranges from 8 × 10^–6^ to 7 × 10^–5^. These values are well within the limit of detection for a backscattering interferometer (BSI) and most analytical-grade conventional RI detectors.^12,54^

Furthermore, when using a 25% increase in polarizability due to the Ca^2+^/Recoverin interaction (e.g., Γ = 1.25) and a 40 Å solvation shell (Figure 1C dark red line), the predicted Δ*n* value matches the experimentally observed B_MAX_ of 3.7 × 10^–5^ RIU (dashed line in Figure 1C). Figure 1C also illustrates that even when using a smaller solvation shell of 30 Å (light orange line) and a small change in *polarizability (ca. 5%)*, the predicted value for Δ*n* is well within the detection limit for BSI (dotted line). Even with small changes in polarizability, relative to those described in the literature,^48^ FreeSRF_CM_ predicts the Ca^2+^/Recoverin interaction to produce a signal well within the sensitivity of both BSI and most scientific grade commercial RI detectors.

Similar calculations were performed using structural information for the benzenesulfonamide binding carbonic anhydrase 2 (CAII). For this pair, literature studies indicate that the shielding of charged residues and ejection of the water molecules results in a decrease in *polarizability,* and therefore a decrease in RI would be predicted.^55,56^ When the unbound benzenesulfonamide (ligand) is free-in-solution, it will have a solvation shell, that upon binding is either released, becoming bulk solvent, or integrated into the receptor’s solvation shell, indicating the use of Γ < 1 values. Figure 1D illustrates that using a solvation shell of 40 Å (blue line) and varying Γ from 0.25 to 0.95 the predicted Δ*n* for the benzenesulfonamide/CAII interaction is predicted by FreeSRF_CM_ to span the range from −1.6 × 10^–6^ to −1.9 × 10^–5^. Note that the predicted ΔRI values are negative, correlating with empirical results.^12^ For a product concentration of 50 nM, the predicted theoretical Δ*n* B_MAX_ is closest to the experimental value of −1.4 × 10^–5^ (Figure 1D dashed line) at a Γ value of 0.42, well within the reasonable range for a change in polarizability.^12^ Even for smaller Γ values (e.g., 10% decrease in total polarizability) and solvation shells of either 40 Å or 30 Å, the model still predicts a quantifiable Δ*n* with respect to the limit of quantification for a benchtop interferometer or standard refractive index detector (Figure 1D dotted line).

## Conculsion

Addressing the need for a revised model for label-free, free-solution interactions, we utilized the Clausius-Mossotti equation and developed a first-principles derivation of a relationship for the solution-phase change in RI. This relationship is the Free solution Response Function (FreeSRF_CM_). We also demonstrated that FreeSRF_CM_ can be used to produce an expression equivalent to our previously published empirical model, FreeSRF_EXP_,^12^ a model predictive of both ΔRI direction and magnitude for a wide range of interactions when using binding induced changes in conformation and hydration.^12^

Next, we showed that using polarizability considerations informed by hydration shell dimensions, FreeSRF_CM_ can be used to calculate the expected range of Δ*n* for two binding systems. By using a range of contemporary literature values for polarizability and solvation shell dimensions it was demonstrated that FreeSRF_CM_ facilitated the accurate prediction of Δ*n* for the Ca^2+^/recoverin and benzenesulfonamide/CAII interactions. When compared to empirical observations performed on a backscattering interferometer (BSI)^12^ it was found there is excellent correlation between the predicted and measured signal magnitudes/directionality. Our results illustrate that free-solution interactions can and *should* produce a quantifiable ΔRI signal that is measurable by, not just interferometric refractometry,^57^ but indeed any sufficiently sensitive RI sensor (∼10^–6^ RIU).

To summarize, the observations reported here stand in stark contrast to those obtained by the currently accepted paradigm (RII)^58,59^ and help to validate the FreeSRF model and the solution-phase measurement methodology. Here we addressed the need for a revised understanding and an improved model for label-free, free-solution chemical/biochemical interactions, with several important variables illuminated by this work. First, it clearly demonstrates a paradigm shift based on first principles. Second, it illustrates the magnitude of ΔRI for solution-phase interactions *should* be and *is* considerably larger than previously assumed or predicted.^58,59^ Third, the binding-induced RI signals that have been widely reported,^13,22,23,60,61^ make physical sense when considering the changes in solution *polarizability.* Fourth, the universal nature of the free-solution measurement,^62 −65^ lack of signal dependence on relative mass and a model based largely on polarizability that is informed by structure indicates the method has potential for wide applicability. Collectively, the FreeSRF model and the solution-phase “platform” represent a new, highly sensitive and informed way to study interactions and could lead to an important and necessary paradigm shift in interaction studies.

While beyond the scope of this report, it would be reasonable to assume that provided the appropriate polarizability information, the FreeSRF model could be applied to interactions in *any* solvent milieu. Examples include observations with BSI on DNA hybridization in mixed solvents^27^ and hydrogen-bonding studies in 100% acetonitrile.^65^ The methodology described here could also lend insight into matrix effects on quantitative assays^8,9^ and provide a better understanding of hydration/structure for membrane-bound vs soluble proteins.^66^

While our current study provides a theoretical framework for refractive index changes in solution-phase molecular interactions, future experimental validation will be crucial to further refine and generalize our model. One potential future experiment would be to perform BSI measurements on the hen egg white lysozyme/buffer system, as modeled in our Supporting Information and previously studied by Ball and Ramsden. Another potential future experiment would be investigating the effects of hydration shell perturbations in water-soluble polymers, such as polyethylene glycol (PEG) or poly(acrylic acid). These polymers offer a controlled system where solvation changes and polarizability shifts can be more easily quantified. By systematically varying polymer concentration and buffer composition, we would be able to study how hydration shell dynamics influence refractive index shifts. Furthermore, using atomistic approaches to estimate monomer-level polarizability would provide a well-defined basis for comparison with our theoretical predictions. Direct experimental correlation of our predicted refractive index changes with empirical measurements would provide additional support for our approach.

The analysis presented herein validates the FreeSRF concept and demonstrates that this approach can be used to estimate the change in solution phase ΔRI for a wide array of solution-phase binding interactions. Under appropriate conditions, this approach could be used to provide important insight into solution phase transitions of nonbinding systems such as protein conformational changes and A-to-B form transition in DNA.^27^ We are currently examining this supposition, and the results of these studies will be presented in due course.

## Supporting Information Available

Extended methods, derivations, Dn/dC changes with conformation and solvation, derivation of the FreeSRF equation from the Clausius–Mossotti relation, and example calculation of RI change using FreeSRF_CM_ (PDF)
