# Supplementary material for: Theoretical Basis for Refractive Index Changes Resulting from Solution Phase Molecular Interaction
Source: J Phys Chem B. 2025 Mar 25;129(13):3297–305. doi: 10.1021/acs.jpcb.4c07563 (PMC11973866; doi:10.1021/acs.jpcb.4c07563)
Supplement: Supplementary file 1 — jp4c07563_si_001.pdf [file jp4c07563_si_001.pdf]

Theoretical Basis for Refractive Index Changes Resulting from Solution Phase Molecular Interaction

Michael N. Kammer†, Amanda K. Kussrow, and Darryl J. Bornhop*

Department of Chemistry and The Vanderbilt Institute of Chemical Biology, Vanderbilt University, Nashville, TN, 37240 USA.

SUPPORTING INFORMATION

Dn/dC changes with conformation and solvation:

In the paper "Buffer Dependence of Refractive Index Increments of Protein Solutions", Ball and Ramsden[1] demonstrated that the experimentally determined RII of hen egg white lysozyme (HEL) varies with the buffer. Their experimentally determined RIIs varied from 0.186 mL/g (a commonly used dn/dC value for proteins[2]) in a buffer containing 100 mM NaCl + 10 mM HEPES to a very large value of 0.272 mL/g in a buffer of 10 mM NaSCN + 10mM HEPES. In nanopure deionized H_2O dn/dC takes on a low value of 0.153 mL/g. Ball and Ramsden attribute the wide range of dn/dC values to different interactions between the solvent and the protein principally due to alterations to the hydrogen bonding within the solvation shell and at the surface of the protein. We can use these values to estimate changes in polarizability or refractive index due solely to changes in conformation and solvation. These values are useful, because they are among the only values in the literature for the RII for a single analyte in multiple solvation states. There is no binding or mass change for this experiment, so any changes must be attributed to solvation effects. While we perform these calculations here with the values from Ball and Ramsden, similar calculations can be performed with the values from Timasheff, Khago, and Hand which result in similar magnitudes for change in refractive index based upon solvent effects. Here we refer to HEL in the 100 mM NaCl + 10 mM HEPES as conformation state I, HEL in the 10 mM NaSCN + 10mM HEPES as conformation state II, and HEL in DI H_2O as state III. Using the molecular weight of HEL (14.3 kDa), we can calculate the change in refractive index between solvent-induced conformations for a chosen concentration of HEL in solution. Using a reasonable concentration typical of a free-solution RI experiment of 50 µM[3-7] produces the results for Δn presented in **Table S1**:

Table S1: RII of proteins depends on conformation/solvation state.

A<br>Conformation/ hydration state	B<br>Buffer	C<br>dn/dc*	D<br>Δn at 50 µM (change from **I**)	E<br>Detection Limit (change from **I**)	F<br>α (Å^3)	G<br>Δα(Å^3)	H Δ α/α
I	NaCl 100 mM + HEPES 10 mM	0.186	-	-	591.6	-	-
II	NaSCN 10 mM	0.272	6.2×10^{-5}	813 nM	865.1	273.5	0.46
III	DI H_2O	0.153	-2.1×10^{-6}	945 nM	486.6	-105.0	-0.12
IV	NaCl 10 mM + HEPES 10 mM	0.188	1.4×10^{-6}	35 µM	598.0	6.4	0.01
V	$NaCH_3COO$ 10 mM + HEPES 10 mM	0.184	-1.4×10^{-6}	35 µM	598.0	-6.4	0.01

*Data taken from Ball and Ramsden 1998

Interestingly, a 50 µM solution of HEL transitioning from state I to state II results in a Δn of 6.2×10^{-5} RIU, which is a relatively large change in RI. Similarly, for the transition between I and III a Δn of -2.1×10^{-6} RIU would be reported. For even a much more conservative change in RII (dn/dC), the transition from the protein in conformation state I to IV or V still results in a Δn of +/-1.4×10^{-6}. Again, these DRI values are in the detectable change by BSI or a scientific grade commercial RI detector.

Taken differently, an instrument with resolution of 10^{-6} RIU, typical for a commercially available refractive index detector, it would be possible to measure the conformational change of HEL transitioning from I to II at a concentration of 813 nM HEL. Even for the more conservative conformational transitions, the result is a detection limit in the low µM range, meaning these interactions would be detectable at physiologically relevant concentrations under conservative estimates.

From the Ball and Ramsden data, we are also able to calculate the effective polarizability of the protein in the various buffers (column F) and therefore the difference in polarizabilities between conformations (column G). Lastly, this analysis allows us to determine the ratio of the change in polarizability to the initial polarizability (column H). The results presented in Table S1 illustrates how significant the change in polarizability is for HEL, which can range from -12% to 46% relative to conformation I. In other words, HEL can undergo a change in polarizability of ca. 50% from solvation effects alone. Thus, a change in polarizability due to solvation effects alone is large

enough to account for the RI change of ~10^{-6}-10^{-5} RIU in some circumstances. When combining solvation effects with the change in polarizability due to molecular rearrangement, or changes in conformation, the *total* change in polarizability for a molecular interaction in free-solution is predicted to be significant in magnitude and 10-1000 times larger than previously predicted.[8] These RI changes are certainly large enough to be detectable by a RI detector with ca.10^{-6} RIU sensitivity.

Derivation of the FreeSRF equation from the Clausius Mossotti Relation:

Equation 7 from the main document:

$$\frac{n_2^2-1}{n_2^2+2}-\frac{n_1^2-1}{n_1^2+2}=\frac{4\pi}{3}\sum_j N_{j2}\alpha_{j2}-\frac{4\pi}{3}\sum_j N_{j1}\alpha_{j1} \qquad \text{Eq. 7}$$

The refractive index of the solution containing the bound complex (n_2) will result from the contribution to the RI from the unbound constituents (n_1) plus the RI change due to the formation of the new complex (Δn): $n_2 = n_1 + \Delta n$.

Approximations: For the range of observed Δn, $1 \times 10^{-7} < \Delta n < 1 \times 10^{-3}$, we can use ${n_2}^2 = {n_1}^2 + \frac{8}{3}\Delta n$ ("Approximation 1" below). This substitution allows us to modify Eq. 3 to give Eq. 4:

$$\frac{n_1^2+\frac{8}{3}\Delta n-1}{n_1^2+\frac{8}{3}\Delta n+2}-\frac{n_1^2-1}{n_1^2+2}=\frac{4\pi}{3}N\cdot\Delta\alpha \qquad \text{Eq. S1}$$

Solving Eq. 4 for Δn results in Eq. 5, a relationship between the change in refractive index and the number density *N* (concentration) and change in polarizability.

$$\Delta n = -\frac{3\pi({n_1}^2+2)^2\cdot(N\cdot\Delta\alpha)}{8\pi({n_1}^2+2)\cdot(N\cdot\Delta\alpha)-18} \qquad \text{Eq. S2}$$

Eq. 7 can now be simplified. First, even for arbitrarily large Δn (10^{-2} RIU), $\Delta\alpha$ remains very small. Consequently, $8\pi(n_1+2)\cdot d\alpha$ $-18 \approx -18$ ("Approximation 2" below). Applying a second simplification for aqueous solutions (n = 1.33 - 1.34), Δn can be estimated to within 1% by substituting 1.333 for n_1 regardless of the true initial RI of the solution before binding (numerically proven under "Approximation 3" below).

Approximation 1:

Even for arbitrarily large Δn (10^{-2} RIU), $d\alpha$ remains very small ($d\alpha < 0.001$). Consequently, ($8\pi(n_1 + 2) \cdot d\alpha - 18 \approx -18$).

Here we will demonstrate that even for arbitrarily large Δn, we can approximate $8\pi(n_1 + 2) \cdot d\alpha - 18 \approx -18$, while obtaining a small (less than 1/10th of 1%) error.

We can rearrange Eq. S2:

$$\Delta n = -\frac{3\pi({n_1}^2 + 2)^2 \cdot (N \cdot \Delta\alpha)}{8\pi({n_1}^2 + 2) \cdot (N \cdot \Delta\alpha) - 18}$$

$$\Delta\alpha(\Delta n * 8\pi({n_1}^2 + 2) \cdot (N) + 3\pi({n_1}^2 + 2)^2 \cdot (N)) = \Delta n * 18$$

To solve for $\Delta\alpha$ in terms of n_1 and Δn:

$$\Delta\alpha = \frac{18\Delta n}{({n_1}^2 + 2) \cdot N \cdot \pi \cdot (8\Delta n + 3)}$$

For arbitrarily large Δn of 0.01 and n_1 = 1.333, $\Delta\alpha$ = 0.0049. Appending Eq. S2 using this approximation provides:

$$\Delta n = \frac{3\pi({n_1}^2+2)^2 \cdot (N \cdot \Delta\alpha)}{18} \quad \text{Eq. S3}$$

Which provides an answer with error <0.01% even at arbitrarily large Δn.

Approximation 2:

$n_2 = n_1 + \mathit{\Delta} n$ is approximately ${n_2}^2 = {n_1}^2 + \frac{8}{3}\mathit{\Delta} n$ in the range of $1 \times 10^{-7} < \mathit{\Delta} n < 1 \times 10^{-3}$

Table S2: Numerical proof for: $n_2 = n_1 + \mathit{\Delta} n \approx \sqrt{{n_1}^2 + \frac{8}{3}\mathit{\Delta} n}$

A) $\boldsymbol{\mathit{\Delta} n}$	B) Exact: $\boldsymbol{n_2 = \sqrt{{n_1}^2 + \frac{8}{3}\mathit{\Delta} n}}$	C) Assumption: $\boldsymbol{n_2 = n_1 + \mathit{\Delta} n}$	D) Error: (C-B)/B
0E+00	1.3330000	1.3330000	0.00%
1E-07	1.3330001	1.3330001	0.03%
2E-07	1.3330002	1.3330002	0.03%
1E-06	1.3330010	1.3330010	0.02%
2E-06	1.3330020	1.3330020	0.02%
1E-05	1.3330100	1.3330100	0.02%
2E-05	1.3330200	1.3330200	0.02%
1E-04	1.3331000	1.3331000	0.02%
2E-04	1.3332000	1.3332000	0.02%
1E-03	1.3340000	1.3339999	0.01%
2E-03	1.3350000	1.3349990	0.05%
1E-02	1.3430000	1.3429653	0.35%

As column D demonstrates, in all cases the error introduced by the assumption is less than 1% of the RI signal (column A). For all cases below $\mathit{\Delta} n = 10^{-2}$, the error is less than 0.1%.

However, without the approximation ${n_2}^2 = (n_1 + \mathit{\Delta} n)^2 \approx {n_1}^2 + \frac{8}{3}\mathit{\Delta} n$, Eq. 8 becomes Eq. S1:

$$\frac{(n_1+\mathit{\Delta} n)^2-1}{(n_1+\mathit{\Delta} n)^2+2} - \frac{n_1^2-1}{n_1^2+2} = \frac{4\pi}{3} N \cdot \Delta\alpha \qquad \text{Eq. S4}$$

In Eq. S1 and S4, $\mathit{\Delta} n$ is in the squared term with n_1, and therefore solving for $\mathit{\Delta} n$ results in a much messier equation:

$$\mathit{\Delta} n = \frac{\sqrt{n_1^2(81-128\cdot\pi^2\cdot N^2\cdot\Delta\alpha^2)-4\pi\cdot N\cdot\Delta\alpha\cdot n_1^4(8\pi\cdot \mathrm{N}\cdot\Delta\alpha+9)+16\pi\cdot N\cdot\Delta\alpha(9-8\pi\cdot N\cdot\Delta\alpha)}-4\pi\cdot N\cdot\Delta\alpha\cdot n_1^3+n_1(9-8\pi\cdot N\cdot\Delta\alpha)}{4\pi\cdot N\cdot\Delta\alpha(n_1^2+2)-9} \qquad \text{Eq. S5}$$

Although Eq. S5 can be evaluated in the same way as Eq. 8 from the main text, it is not as easily interpreted, and it offers little improvement to the calculated $\mathit{\Delta} n$ (less than 0.5% at arbitrarily large $\mathit{\Delta} n$).

Approximation 3:

For aqueous solutions (n = 1.33-1.34), Δn can be estimated to within 1% by substituting 1.333 for n_1 regardless of the true initial refractive index of the solution before binding.

A) n_1	B) Exact $\Delta n = \frac{3\pi}{18}(n_1^2+2)^2 \cdot N \cdot \Delta\alpha$	C) Assumption $\Delta n = \frac{3\pi}{18}(1.333^2+2)^2 \cdot N \cdot \Delta\alpha$	D) Error $\frac{C-B}{B}$
1.330	0.000581	0.000582	0.18%
1.331	0.000581	0.000582	0.12%
1.332	0.000581	0.000582	0.06%
1.333	0.000582	0.000582	0.00%
1.334	0.000582	0.000582	0.06%
1.335	0.000582	0.000582	0.12%
1.336	0.000583	0.000582	0.18%
1.337	0.000583	0.000582	0.24%
1.338	0.000583	0.000582	0.30%
1.339	0.000584	0.000582	0.36%
1.340	0.000584	0.000582	0.42%

The refractive index of the bulk solvent (n_1) has a small effect on the final calculated Δn, allowing us to use this approximation (n_1 = 1.333), greatly reducing the complexity of the equation. Using the assumption $n_1 = 1.333$ results in a final error of less than half a percent when compared to using the full version. This assumption enables the $\frac{3\pi}{18}(1.333^2+2)^2$ term to be taken as a constant ~7.47, simplifying the equation. When performing calculations computationally, the exact value for n_1 can be used.

When performing calculations for systems in non-aqueous solutions, such as binding systems in organic solvents, the RI of the organic solvent can be used in place of n_1.

Example Calculation of RI change using FreeSRF$_{CM}$: Carbonic anhydrase II (CAII) - benzene sulfonamide

First, we estimate the polarizability of the unbound protein receptor, using an amino-acid composite model. Here the polarizability of each amino acid in the protein sequence was summed,[9] using values for electronic polarizabilities for amino acids were taken from Voges and Karshikoff.[10] Using the Voges approach, which has been demonstrated to provide a reasonable starting point for molecular polarizability and refractive index increment (RII) estimates,[11,12] we calculated the polarizability of carbonic anhydrase to be 3,045 Å^3.

Next, the polarizability of the ligand is calculated. It is noteworthy that since all of the ligands studied here were either small molecules or ions, we estimated their polarizability by using an atom-based composite model.[13] In this model the polarizabilities of each atom are summed. Using the atom-based approach yields a polarizability for benzene sulfonamide of 116 Å^3.

Calculation of the polarizability of the ligand, receptor and the product solvation shells: their volume is determined in UCSF Chimera by using protein structures obtained from the RCSB Protein Databank.[14] Second, the number of waters in the solvation shell is calculated by dividing the total volume of the solvation shell by the volume of a molecule of water. Third, the number of waters is multiplied by the polarizability of the water molecule (7, 8). From this determination we obtain the polarizability of the solvation shell of the ligand and the receptor.

For example, to calculate the volume of the receptor (ligand determination follows similarly) we used a solvation shell dimension of 30 Å and Chimera, which gives a total volume of 931,733 $Å^3$. Then after subtracting the protein volume (32,910 Å3 from the PDB structure for unbound carbonic anhydrase II, 1CA1) from the total volume of 931,733 $Å^3$, the solvation shell volume for the receptor CAII of 898,823 $Å^3$ is obtained (931,733 - 32,910 = 898,823). From this quantity the number of waters in the solvation shell (~36,000) is found by dividing the volume of solvation shell by the volume of a water molecule (25 $Å^3$). Using the average polarizability of a solvation-shell water molecule of 1.5 $Å^3$ [15,16] results in a polarizability for the solvation shell of 53,929 $Å^3$. Combining terms gives a 'total' polarizability of the unbound CA II, with a 30 Å solvation shell, of 56,975 $Å^3$.

Using similar arguments, data from the PDB and Chimera, the 'total' polarizability of the ligand, plus its solvation shell is calculated to be 9,539 $Å^3$. For convenience the polarizability can be expressed in volume (Liters) by using the relationship that 1 L = 10^{27} $Å^3$.

Figure S1. Example workflow for polarizability calculation.

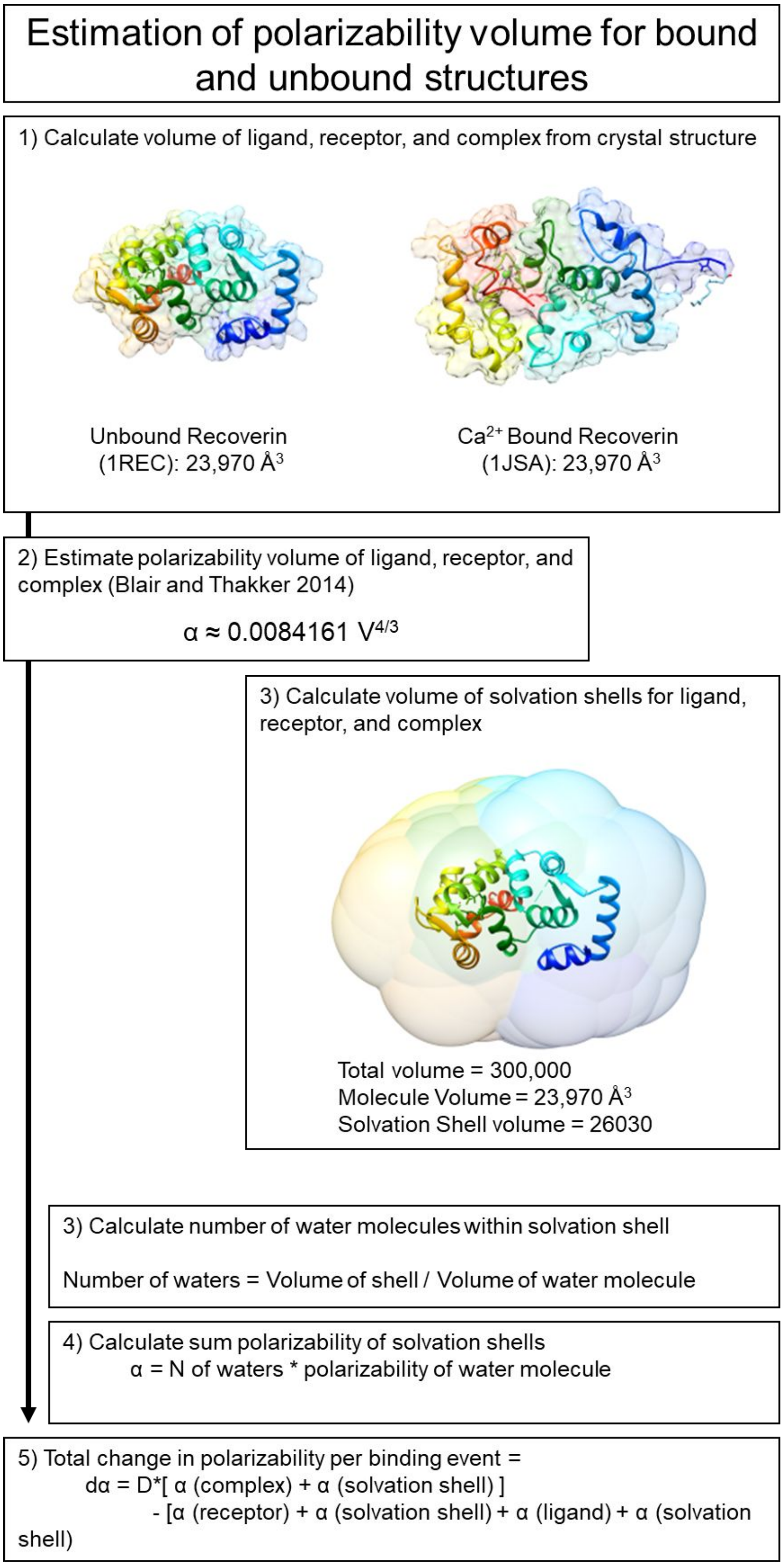

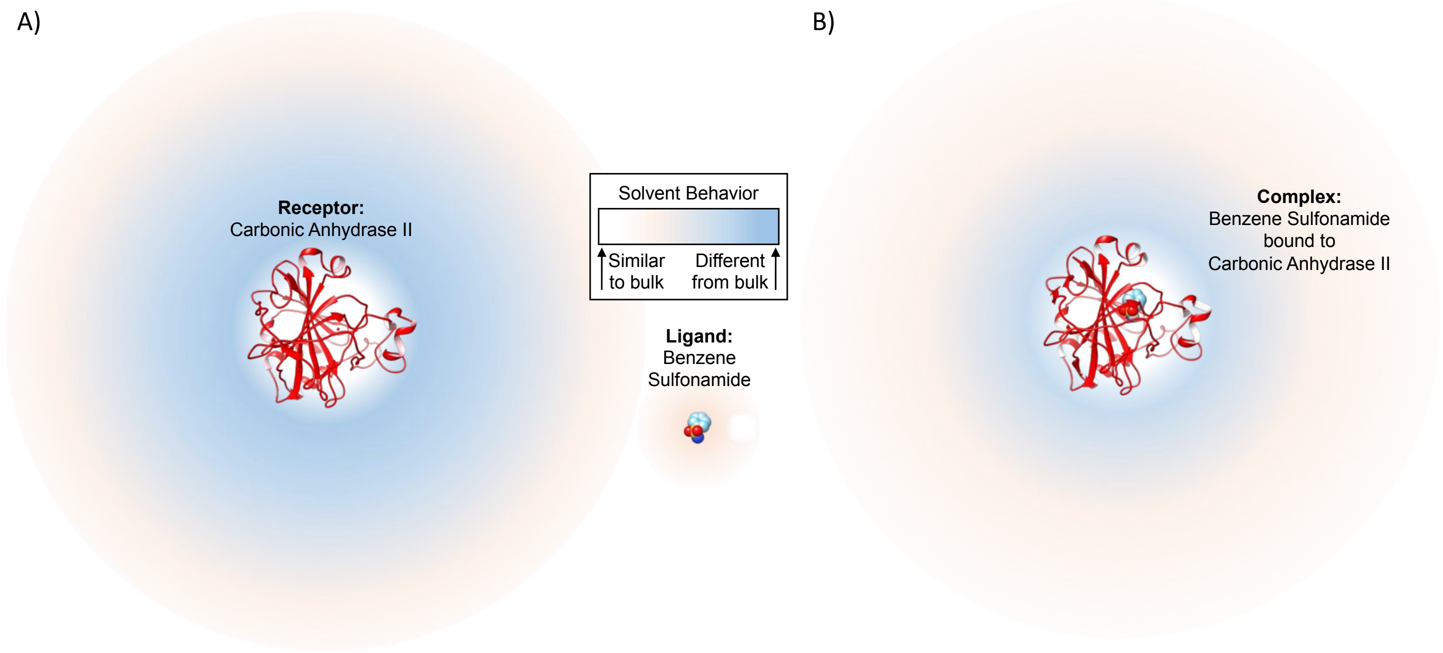

Figure S2. The unbound receptor perturbs the solvent around it (the solvation shell). This perturbation is more pronounced near the protein and diminishes with the distance from the protein (blue shaded area). The unbound ligand perturbs the solvent in a similar way. The total effective dielectric constant of the receptor in solution is the sum of its own dielectric constant and the dielectric of the solvation shell. **B.** When the ligand and receptor form a complex, both the distribution of perturbation of the solvation shell and the dielectric distribution of the solute changes.

References

[1] V. Ball and J. J. Ramsden, "Buffer dependence of refractive index increments of protein solutions," *Biopolymers,* vol. 46, pp. 489-492, Dec 1998.

[2] H. Zhao, P. H. Brown, and P. Schuck, "On the Distribution of Protein Refractive Index Increments," *Biophysical Journal,* vol. 100, pp. 2309-2317, 2011.

[3] M. N. Kammer, A. K. Kussrow, R. L. Webster, H. D. Chen, M. Hoeksema, R. Christenson, P. P. Massion, and D. J. Bornhop, "Compensated Interferometry Measures of CYFRA 21-1 Improve Diagnosis of Lung Cancer," *Acs Combinatorial Science,* vol. 21, pp. 465-472, Jun 2019.

[4] C. L. Retzlaff, A. Kussrow, T. Schorkopf, P. Saetear, D. J. Bornhop, J. A. Hardaway, S. M. Sturgeon, J. Wright, and R. D. Blakely, "Metallo-beta-lactamase Domain-Containing Protein 1 (MBLAC1) Is a Specific, High-Affinity Target for the Glutamate Transporter Inducer Ceftriaxone," *Acs Chemical Neuroscience,* vol. 8, pp. 2132-2138, Oct 2017.

[5] M. M. Wang, A. K. Kussrow, M. F. Ocana, J. R. Chabot, C. S. Lepsy, D. J. Bornhop, and D. M. O'Hara, "Physiologically relevant binding affinity quantification of monoclonal antibody PF-00547659 to mucosal addressin cell adhesion molecule for in vitro in vivo correlation," *British Journal of Pharmacology,* vol. 174, pp. 70-81, Jan 2017.

[6] M. N. Kammer, I. R. Olmsted, A. K. Kussrow, M. J. Morris, G. W. Jackson, and D. J. Bornhop, "Characterizing aptamer small molecule interactions with backscattering interferometry," *Analyst,* vol. 139, pp. 5879-5884, Nov 21 2014.

[7] A. Kussrow, C. S. Enders, and D. J. Bornhop, "Interferometric Methods for Label-Free Molecular Interaction Studies," *Analytical Chemistry,* vol. 84, pp. 779-792, Jan 17 2012.

[8] M. M. Varma, "Article does not explain the origin of free-solution protein interaction signals," *Proceedings of the National Academy of Sciences of the United States of America,* vol. 113, pp. E4930-E4930, Aug 23 2016.

[9] H. Y. Zhao, P. H. Brown, and P. Schuckt, "On the Distribution of Protein Refractive Index Increments," *Biophysical Journal,* vol. 100, pp. 2309-2317, May 4 2011.

[10] D. Voges and A. Karshikoff, "A model of a local dielectric constant in proteins," *The Journal of Chemical Physics,* vol. 108, pp. 2219-2227, 1998.

[11] S. W. Wang, Y. Z. Shao, E. Alexov, and S. Zhao, "A regularization approach for solving the super-Gaussian Poisson-Boltzmann model with heterogeneous dielectric functions," *Journal of Computational Physics,* vol. 464, Sep 1 2022.

[12] A. S. Bayden, M. Fornabaio, J. N. Scarsdale, and G. E. Kellogg, "Web application for studying the free energy of binding and protonation states of protein-ligand complexes based on HINT," *Journal of Computer-Aided Molecular Design,* vol. 23, pp. 621-632, Sep 2009.

[13] B. T. Thole, "Molecular polarizabilities calculated with a modified dipole interaction," *Chemical Physics,* vol. 59, pp. 341-350, 1981/08/01/ 1981.

[14] H. M. Berman, J. Westbrook, Z. Feng, G. Gilliland, T. N. Bhat, H. Weissig, I. N. Shindyalov, and P. E. Bourne, "The Protein Data Bank," *Nucleic Acids Research,* vol. 28, pp. 235-242, Jan 1 2000.

[15] B. Schropp and P. Tavan, "The Polarizability of Point-Polarizable Water Models: Density Functional Theory/Molecular Mechanics Results," *The Journal of Physical Chemistry B,* vol. 112, pp. 6233-6240, 2008/05/01 2008.

[16] M. Akihiro, "Water polarizability in condensed phase: Ab initio evaluation by cluster approach," *Journal of Computational Chemistry,* vol. 23, pp. 1466-1471, 2002.
